# Severe kidney dysfunction after assisted reproductive technology: a case series suggesting the need for higher awareness of risks

**DOI:** 10.1007/s40620-025-02226-4

**Published:** 2025-02-07

**Authors:** Eman Nagy, Rasha Shemies, Mohamed Taman, Nagy Sayed-Ahmed, Giorgina Barbara Piccoli

**Affiliations:** 1 Mansoura Nephrology and Dialysis Unit, Internal Medicine Department, Faculty of Medicine, Mansoura University, Mansoura, Egypt; 2 Department of Obstetrics and Gynecology, Faculty of Medicine, Mansoura University, Mansoura, Egypt; 3 Néphrologie, Centre Hospitalier Le Mans, Le Mans, France

**Keywords:** Assisted reproductive technology, Pregnancy, Acute kidney injury, Chronic kidney disease

## Abstract

**Background:**

Assisted reproductive technology (ART) has significantly increased the rate of conception and live births in women with fertility problems. Having a kidney disease negatively affects a woman’s reproductive health, making infertility a significant concern, and women with chronic kidney disease (CKD) are increasingly seeking treatment with assisted reproductive technology. The side effects of assisted reproductive technology are not fully known and its impact on kidney function, especially if a woman has undergone repeated treatments, is likewise not known.

**Methods:**

This case series gathers all consecutive patients who were followed by the Mansoura University Hospital’s Obstetric Nephrology Service or were admitted to its Nephrology and Gynecology Department during pregnancy with a diagnosis of acute or chronic kidney function impairment after conceiving with an assisted reproductive technology method, in the period from 2021 to 2024.

**Results:**

Of the approximately 150 pregnancies referred to the Obstetric Nephrology Clinic, 6 were referred for acute or acute-on-chronic kidney function impairment, or nephrotic syndrome after conceiving via assisted reproductive technology. In one patient, CKD was overlooked and later progressed to kidney failure; one had probable CKD, but discontinued follow-up before confirmation; and one had a kidney malformation, diagnosed during pregnancy. All presented with early or very early severe hypertension and proteinuria, before 20 weeks, while preeclampsia and the hypertensive disorders of pregnancy are conventionally defined as developing after 20 weeks of gestation. Three had complete recovery postpartum, one progressed to kidney failure, while two were lost to follow-up.

**Conclusion:**

Severe early-pregnancy kidney impairment after assisted reproductive technology is probably more frequent than previously reported. Assessment of kidney function after assisted reproductive technology should be mandatory, to make it possible for timely specialized kidney care to be given*.*

**Graphical abstract:**

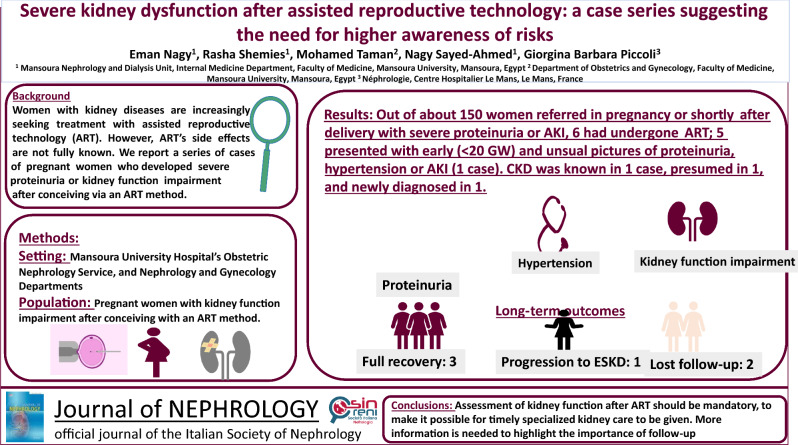

## Background

Reproductive freedom means guaranteeing that every woman, whether healthy or affected by a disease, has the right to make decisions concerning her fertility [1]. This acknowledged human right should not have borders. Large families are a priority in some cultural contexts, for example in Egypt, where this case series was described [2]. Assisted reproductive technology (ART), which makes it possible for otherwise infertile couples to have children, is extremely important, but also exposes those seeking treatment to economic exploitation and sometimes to uncontrolled procedures. Assisted reproductive technology refers to a range of treatments addressing infertility. Following the first report on human assisted reproductive technology in 1943 [3], and the first successful human in vitro fertilization (IVF) performed by Robert G. Edwards and Patrick Steptoe in 1978 [4], by 2019 assisted reproductive technology had made possible over 8 million live births [5].

There are several assisted reproductive technology techniques, including ovulation induction and intrauterine insemination, but IVF, and intracytoplasmic sperm injection (ICSI) are currently the most widely used. They are preceded by the induction of ovulation, which entails the administration of oral or injectable gonadotropins in the first phase of the ovarian cycle. Following the induction of ovulation, oocytes are retrieved from the ovary using needle aspiration; in IVF, oocytes are incubated with sperm in vitro [6], while in intracytoplasmic sperm injection, a spermatocyte is injected directly into the cytoplasm of an oocyte.

All types of assisted reproductive technologies are associated with an increase in the incidence of hypertensive disorders of pregnancy, from severe pregnancy-induced hypertension to preeclampsia (PE) and hemolysis, elevated liver enzymes, and low platelet count (HELLP) syndrome, and the risks increase in parallel with the increasing complexity of the procedure employed [7–9]. The specific role of hormonal conditioning, common to most assisted reproductive technologies is difficult to assess but may be relevant, in particular in the presence of baseline risk factors, such as hypertension, obesity, and CKD [10, 11]. However, the effect assisted reproductive technology techniques has on kidney function has not been studied, and only occasional reports describe atypical presentations of hypertensive disorders of pregnancy after assisted reproductive technology treatment, occasionally with severe kidney involvement [12].

It is well known that CKD, in particular in its late stages, is associated with lower fertility rates when compared to the general population, a problem partly shared by kidney transplant recipients (KTRs) [13]. While women with kidney diseases often decide to undergo treatment with assisted reproductive technology so they can become pregnant, data regarding the safety of assisted reproductive technology treatment in women with CKD are scant. The conclusions of a recent systematic review and meta-analysis of assisted reproductive technology in women with CKD were optimistic, but the high success rate in this small number of cases, most of whom were treated after kidney transplantation, raises the suspicion of relevant reporting bias [14]. The fact that kidney function matters is also suggested by an analysis of the relationship between serum creatinine and materno-fetal outcomes after egg donation [15].

In this article, we describe 6 women who became pregnant after an assisted reproductive technology treatment, experienced severe renal complications, in some cases with fetal loss, and were referred to our clinic in Mansoura, the first specialized facility in Egypt dedicated to kidney disease during pregnancy.

## Methods

### Study setting

The six cases reported were observed over a relatively short period of time (2021–2024) in the Obstetric Nephrology Clinic of the Mansoura University Hospital in Egypt. Since the start of its activity in 2021, the main clinical data of all patients referred to the CKD-pregnancy clinic have been recorded and archived in a computerized database (about 150 pregnancies at the time of the present report). Data were collected from clinical charts and reviewed by the senior local author (RS); all cases were discussed with an external expert (GBP). Of note, in the same period six further cases were referred to the obstetric ward. However, in the absence of quantification of proteinuria, and given that detailed data on the patients’ clinical history were lacking, they were not included in this series.

## Assisted reproduction techniques

The most commonly used protocol for assisted reproductive techniques in our setting is the antagonist protocol where ovulation induction is initiated on the second day of menstruation using a dose of 150–225 IU of gonadotropin (mainly recombinant FSH) adjusted according to the patient’s BMI, and administered for 6 days. Subsequently, the size of growing follicles is measured using transvaginal ultrasound. On the 6th day of stimulation, a gonadotropin-releasing hormone (GnRH) antagonist (cetrorelix 0.25 mg daily) is added to suppress the hypothalamic-pituitary-ovarian axis in association with gonadotropins containing medications. When at least 3 leading follicles reach a size of more than 17 mm in diameter, human chorionic gonadotropin (HCG 10000 IU) or a GnRH agonist (triptorelin 0.2–0.3 mg) is given as a single shot to induce the maturation of oocytes and trigger ovulation. Oocyte retrieval is performed 34–36 h after the trigger, after which either IVF or intracytoplasmic sperm injection is performed. According to the quality and the number of the formed embryos, embryo transfer is planned after 3 days (cleavage stage) or 5 days (blastocyst stage) of oocyte retrieval. Micronized progesterone (400 mg vaginal suppositories) is given twice daily as luteal phase support until a serum pregnancy test and transvaginal ultrasound are performed 14 days after embryo transfer.

## Results

The main clinical data on the 6 cases are reported in Table 1. All but one had undergone previous assisted reproductive technology treatment, up to 7 times in case 2. All had undergone assisted reproductive technology in a private setting, and the details of the procedures were not available; serum creatinine preconception was available for only one woman. In 5 cases the onset of symptoms was very early, before the 20th gestational week, while it was at 25 weeks in the remaining woman, who had acute kidney injury (AKI) and a twin-to-twin transfusion syndrome. None of the patients followed up to delivery was able to leave the hospital “baby in arms”; the only live-born baby died a few days after delivery. An indication of the many barriers that exist to providing care, is that two patients, one during pregnancy, were lost to follow-up.

**Table 1:** Summary of the 6 cases observed in the nephrology unit

	Case 1	Case 2	Case 3	Case 4	Case 5	Case 6
Age (years)	36	27	29	26	24	37
Fetuses	Twin	Single	Single	Twin	Twin	Single
Previous pregnancies	1 miscarriage	No	No	No	1 miscarriage	1 intrauterine death
Previous treatments with ART	1	7	2	None	1	1
Associated comorbidity	Obesity and hypothyroidism	Hypothyroidism	FSGS and DM type 1	History of proteinuria	No	Hypertension
Type of ART	ICSI	ICSI	IVF	IVF	ICSI	ICSI
Setting of ART	Private	Private	Private	Private	Private	Private
Protocol for controlled ovarian stimulation	Not available	Not available	Not available	Not available	Not available	Not available
Low dose aspirin /heparin	Yes	Yes	Yes	Yes	Yes	Yes
Serum creatinine before conception (mg/dL)	Not known	Not known	1.2	Not known	Not known	Not known
Serum creatinine at referral (mg/dL)	0.8	0.6	1.5	0.7	1.9	0.6
Proteinuria at referral (g/day)	2.4	12	3.5	3.5	0.58	1.7
Serum creatinine after delivery (mg/dL)	0.6	0.6	1.4	0.9	0.9	0.6
Proteinuria after delivery (mg/day)	162	123	560	3200	Not known	Not known
Gestational age at first symptoms (GW)	17	18	6	8	25	19
Gestational age at delivery-termination of pregnancy	24	24	26	9	25	Lost to follow-up in pregnancy
Fetal outcome	Stillbirth	Stillbirth	Neonatal death	Miscarriage	Stillbirth, neonatal death	Lost to follow-up during pregnancy
Maternal outcome	Kidney function recovery	Kidney function recovery	Kidney failure 2 years later; dialysis	Lost to follow-up	Kidney function recovery	Lost to follow-up

*ART* assisted reproductive technology, *ICSI* intracytoplasmic sperm injection, *GNRH* gonadotropin-releasing hormone, *IVF* in vitro fertilization

## Detailed information on the cases

### Patient 1

A 36-year-old woman with obesity (BMI 35.7 kg/m^2^), a history of hypothyroidism, now under treatment, was referred at 17 gestational weeks (GW) because of proteinuria and hypertension. Her first pregnancy was obtained by intracytoplasmic sperm injection six months previously, with a miscarriage at 6 gestational weeks. The current pregnancy was obtained via a second intracytoplasmic sperm injection, that resulted in a twin pregnancy, but one fetus underwent early spontaneous abortion (6 weeks). On presentation, her blood pressure was 150/90 mmHg, serum creatinine was 0.8 mg/dL, and 24 h proteinuria was 2.4 g. Serum albumin was 3.3 g/dl, and antinuclear antibody (ANA) and anti-double-stranded deoxyribonucleic acid antibodies (anti-dsDNA) were both negative. Her thyroid profile was normal. Medications included aspirin and enoxaparin (prescribed after the assisted fertilization procedure), alpha methyl dopa, and nifedipine. At 21 gestational weeks hypertension control worsened, and progressive edema developed. At 22 gestational weeks, a kidney biopsy was performed but did not lead to a definitive diagnosis: it revealed non-specific thickening of the basement membrane without spikes or vacuolization, along with mesangial accentuation but no hypercellularity. Intrauterine fetal growth restriction (IUGR) was evident at 22 gestational weeks, followed by intrauterine death at 24 gestational weeks. Four weeks postpartum, the patient had normal blood pressure without medications, edema resolved, and her 24 h proteinuria was 780 mg. One year later, at the last follow-up, her serum creatinine was 0.64 mg/dL and proteinuria was in the normal range (16.2 mg/day). In retrospect, complete recovery without treatment is in line with the lack of specific lesions at kidney biopsy.

### Patient 2

A 27-year-old woman who had previously undergone 7 unsuccessful cycles of intracytoplasmic sperm injection/IVF was referred during her first pregnancy because of hypertension and proteinuria, discovered at 18 gestational weeks. At the time of referral (22 gestational weeks), her blood pressure was 150/100 mm/Hg despite three antihypertensive medications (labetalol, methyldopa, and nifedipine). Her serum albumin was 2.5 g/dL, serum creatinine was 0.6 mg/dL, and 24 h urinary proteinuria was 12 g/day. In spite of a history of hypothyroidism, her thyroid and immunological profiles were normal. Notably, at 23 weeks, soluble fms-like tyrosine kinase 1 (sFlT) was 26.156 pg/mL, and placental growth factor (PlGF) was < 10 pg/mL. The tests were performed outside the country and intrauterine fetal death was diagnosed by the time results were available. Six weeks postpartum, the patient had complete normalization of blood pressure and laboratory data.

### Patient 3

A 29-year-old woman affected by type 1 diabetes mellitus since childhood, with a histologic diagnosis of “primary” focal segmental glomerulosclerosis (FSGS) at the age of 17, was referred to our clinic at 6 gestational weeks, with hypertension, oliguria and kidney function impairment; her proteinuria was 3.5 g/day and her serum creatinine 1.5 mg/dl. Her kidney function had previously been normal, and proteinuria had been absent for 10 years. The current pregnancy was obtained using IVF, following two unsuccessful IVF trials. Treatment with prednisolone (60 mg) and cyclosporine (75 mg) was started in the hypothesis of an FSGS flare. However, on maximum dosage of alpha-methyl-dopa and nifedipine, proteinuria continued to increase, and hypertension control was poor. Despite the challenging situation, she insisted on continuing her pregnancy. At 26 weeks of gestation, she experienced preterm premature rupture of membranes and delivered a female baby, who died in the neonatal ICU. After delivery, the patient showed an initial improvement in kidney function but subsequently progressed to kidney failure and was started on chronic hemodialysis two years after delivery.

### Patient 4

A 26-year-old woman with a six-month history of hypertension presented one week after the spontaneous abortion of her twin fetuses, occurring at 12 gestational weeks. Pregnancy was obtained by IVF, and hypertension (160/100 mmHg) and proteinuria (proteinuria 3.8 g/d, serum albumin 3.1 g/dL) with edema developed in the 10th gestational week. Serum creatinine was within the normal range. The patient reported a vague history of having abnormal urinary proteins before IVF, but she declined a kidney biopsy and missed her follow-up appointment.

### Patient 5

A 24-year-old woman was referred because of bilateral lower limb edema and oliguria in the 25th week of gestation of a twin pregnancy obtained by intracytoplasmic sperm injection. At referral, her blood pressure was 110/70 mm/Hg. The workup revealed a left pelvic kidney and normal right kidney, increased serum creatinine (1.8 mg/dL), serum albumin of 3.5 g/dL, and 24-h urinary proteinuria was 580 mg/day. On admission, and in view of the unavailability of other treatment options, a decision of pregnancy termination was made by the obstetrics team based on the likelihood of a twin-to-twin transfusion syndrome, which resulted in the death of one of the fetuses. The live-born baby was admitted to the neonatal ICU but died a few days later. After delivery, the patient’s kidney function improved, and serum creatinine reached normal levels (0.9 mg/dL) four weeks after delivery.

### Patient 6

A 37-year-old woman presented to the obstetrics emergency room at 19 weeks of gestation in her second pregnancy, obtained by intracytoplasmic sperm injection, with severe hypertension (blood pressure 180/110 mm/Hg). She reported a 10-year history of well-controlled hypertension. Her previous pregnancy, also conceived via intracytoplasmic sperm injection, resulted in preeclampsia and intrauterine fetal death. The patient had not been fully compliant with antihypertensive treatment (nifedipine and alpha methyl dopa) started early in pregnancy. Additionally, she had been prescribed low-molecular-weight heparin and maintained on low-dose prednisone for unsubstantiated indications since the early weeks of this pregnancy. Laboratory results showed proteinuria of 1.7 g/day, serum creatinine of 0.6 mg/dL and serum albumin of 3.3 g/dL. Liver function tests were normal. Immunologic work-up, including tests for anti-phospholipid syndrome, were negative. After her blood pressure was stabilized, she was discharged from the obstetrics department. She subsequently missed her follow-up appointments.

## Discussion

Assisted reproductive techniques are associated with a higher incidence of hypertensive disorders of pregnancy, partly explained by multiple gestations, advanced maternal age, comorbidities, cause of infertility, and ovarian hyperstimulation syndrome [16, 17]. Kidney impairment can be expected to be common in such a setting, considering its association with lower fertility rates, but may go unreported, particularly in medium–low resource settings, due to a lack of awareness of the importance of kidney function tests and absence of screening protocols.

The present study reports six cases of severe proteinuria, with hypertension and kidney function impairment occurring after different assisted reproductive technology procedures, five in the context of early-onset hypertensive disorders of pregnancy, and one with acute kidney injury. This series exemplifies threatening situations that could, at least in part, be prevented, and whose risk should be considered and explained before women begin assisted reproductive technology procedures.

Only one of our patients had undergone kidney function tests before assisted reproductive technology, and a history of previous kidney disease (biopsy-proven FSGS) was overlooked in one patient (case 3); CKD may have been present in another patient (case 4) who was, however, lost to follow-up before diagnosis was completed. Conversely, in case 5 a diagnosis of pelvic kidney was made, which should have led to stricter follow-up during pregnancy, for the well-known association between all renal alterations, including interstitial diseases, and higher risk of adverse pregnancy outcomes [18]. While kidney function assessment prior to assisted reproductive technology is not mandatory, some authors hold that kidney function assessment should be considered in all pregnancies [19, 20] or at least in high-risk situations, including assisted reproductive technology (the 2023 KDIGO Conference in Athens). None of our patients had been referred for preconception counseling, and a measure of the lack of information that exists can also be seen in the difficulty we had in retrieving information on their medical histories, treatments and procedures (Table 1).

The most important finding in this series is the early timing of onset of proteinuria and hypertension, occurring before the classic term of 20 gestational weeks, identified as diagnosing hypertensive disorders of pregnancy. While exceptions exist, and some have been reported, especially in multiple pregnancies or after oocyte donation, to date this is the largest series reporting on severe kidney involvement after assisted reproductive technology, in the context of conjoint nephrology and obstetric management [21, 22]. Interestingly, only three of our cases were twin pregnancies and none had undergone oocyte donation. A recent systematic review, published in 2023 [23], reported on 37 cases of early-onset preeclampsia, only 10% of which ended in live births. In this series, 6 were multiple pregnancies, 9 partial or complete molar pregnancies, and the use of an assisted reproductive technology was reported in 3 cases (two egg donations). Of note, in 7 cases reported in this review anti-phospholipid antibodies were present, indicating the close relationship between pregnancy and autoimmune and kidney diseases, and the difficulties encountered in diagnosing hypertensive disorders of pregnancy in these settings [23].

The diagnosis of hypertensive disorders of pregnancy is clinical and is supported by at least initial improvement in the 5 cases followed up after delivery, with full remission in three. The availability of placental biomarkers, that were however tested in only one case, and outside our country, could help in these situations by supporting a differential diagnosis between CKD and preeclampsia. This highlights how low availability of diagnostic tools (and potential treatments) is a barrier that needs to be overcome in low-medium income countries and underlines that kidney involvement needs to be given greater attention. Likewise, an important limitation is that information on our cases was often incomplete, an issue that is shared by the studies done in developing countries, in particular when private medicine is involved.

A further important suggestion comes from the observation that in our series only one woman was undergoing assisted reproductive technology for the first time, while the other five had undergone as many as 7 previous attempts of assisted fertilization. Within the limits of our report, this suggests that greater attention should be paid to kidney function in women who have undergone several assisted reproductive technology cycles, an issue as yet unexplored.

Caring for these patients poses not only clinical, but also ethical questions, that remain, for the moment, unsolved. None of the women we describe had a living child, although all of them had been married for several years, and they all desperately wanted to give birth. We have no data that would allow us to advise them against trying to conceive again, even if our experience on the recurrence of hypertensive disorders of pregnancy supports concerns for their kidney function and overall health, should they once more undergo assisted reproductive technology [24–26].

In conclusion, kidney dysfunction is a potential, possibly underreported complication, after assisted reproductive technology protocols, especially if repeated and if performed outside carefully monitored situations.

The association between assisted reproductive technology and early onset hypertensive disorders of pregnancy remains an area requiring further investigation as published data are insufficient to explore this association and well-designed future studies are needed to further elucidate our observation.

Especially in low-medium income countries, in which cultural pressure to have large families is strong, and being childless may be seen as a stigma, the risks connected with assisted reproductive technology can be particularly challenging. Better care, more resources, and better health education are still unmet needs. Health equity still has a long way to go when pregnancy is concerned.

## Data Availability

The data used are available from the corresponding author on reasonable request.
